# A Paradigm for Virus–Host Coevolution: Sequential Counter-Adaptations between Endogenous and Exogenous Retroviruses

**DOI:** 10.1371/journal.ppat.0030170

**Published:** 2007-11-09

**Authors:** Frederick Arnaud, Marco Caporale, Mariana Varela, Roman Biek, Bernardo Chessa, Alberto Alberti, Matthew Golder, Manuela Mura, Ya-ping Zhang, Li Yu, Filipe Pereira, James C DeMartini, Kreg Leymaster, Thomas E Spencer, Massimo Palmarini

**Affiliations:** 1 Institute of Comparative Medicine, University of Glasgow Veterinary School, Glasgow, Scotland; 2 Division of Environmental and Evolutionary Biology, Institute of Biomedical and Life Sciences, University of Glasgow, Glasgow, Scotland; 3 Sezione di Malattie Infettive del Dipartimento di Patologia e Clinica Veterinaria, University of Sassari, Sassari, Italy; 4 State Key Laboratory of Genetic Resources, Kunming Institute of Zoology, Chinese Academy of Sciences, Kunming, China; 5 Laboratory for Conservation and Utilization of Bioresources, Yunnan University, Kunming, China; 6 Instituto de Patologia e Imunologia Molecular da Universidade do Porto, Porto, Portugal; 7 Faculdade de Ciências, Universidade do Porto, Porto, Portugal; 8 Department of Microbiology, Immunology and Pathology, Colorado State University, Fort Collins, Colorado, United States of America; 9 United States Meat Animal Research Center, Clay Center, Nebraska, United States of America; 10 Center for Animal Biotechnology and Genomics, Department of Animal Science, Texas A&M University, College Station, Texas, United States of America; King's College London, United Kingdom

## Abstract

Endogenous retroviruses (ERVs) are remnants of ancient retroviral infections of the host germline transmitted vertically from generation to generation. It is hypothesized that some ERVs are used by the host as restriction factors to block the infection of pathogenic retroviruses. Indeed, some ERVs efficiently interfere with the replication of related exogenous retroviruses. However, data suggesting that these mechanisms have influenced the coevolution of endogenous and/or exogenous retroviruses and their hosts have been more difficult to obtain. Sheep are an interesting model system to study retrovirus-host coevolution because of the coexistence in this animal species of two exogenous (i.e., horizontally transmitted) oncogenic retroviruses, Jaagsiekte sheep retrovirus and Enzootic nasal tumor virus, with highly related and biologically active endogenous retroviruses (enJSRVs). Here, we isolated and characterized the evolutionary history and molecular virology of 27 enJSRV proviruses. enJSRVs have been integrating in the host genome for the last 5–7 million y. Two enJSRV proviruses (enJS56A1 and enJSRV-20), which entered the host genome within the last 3 million y (before and during speciation within the genus *Ovis*), acquired in two temporally distinct events a defective Gag polyprotein resulting in a transdominant phenotype able to block late replication steps of related exogenous retroviruses. Both transdominant proviruses became fixed in the host genome before or around sheep domestication (∼ 9,000 y ago). Interestingly, a provirus escaping the transdominant enJSRVs has emerged very recently, most likely within the last 200 y. Thus, we determined sequentially distinct events during evolution that are indicative of an evolutionary antagonism between endogenous and exogenous retroviruses. This study strongly suggests that endogenization and selection of ERVs acting as restriction factors is a mechanism used by the host to fight retroviral infections.

## Author Summary

The genome of all vertebrates is heavily colonized by “endogenous” retroviruses (ERVs). ERVs derive from retrovirus infections of the germ cells of the host during evolution, leading to permanent integration of the viral genome into the host DNA. Because ERVs are integrated in the host genome, they are transmitted to subsequent generations like any other host gene. The function of endogenous retroviruses is not completely clear, but some ERVs can block the replication cycle of horizontally transmitted “exogenous” pathogenic retroviruses. These observations lead to the hypothesis that ERVs have protected the host during evolution against incoming pathogenic retroviruses. Here, by characterizing the evolutionary history and molecular virology of a particular group of endogenous betaretroviruses of sheep (enJSRVs) we show a fascinating series of events unveiling the endless struggle between host and retroviruses. In particular, we discovered that: (i) two enJSRV loci that entered the host genome before speciation within the genus *Ovis* (∼ 3 million y ago) acquired, after their integration, a mutated defective viral protein capable of blocking exogenous related retroviruses; (ii) both these transdominant enJSRV loci became fixed in the host genome before or around sheep domestication (∼ 10,000 y ago); (iii) the invasion of the sheep genome by ERVs of the JSRV/enJSRVs group is still in progress; and (iv) new viruses have recently emerged (less than 200 y ago) that can escape the transdominant enJSRV loci. This study strongly suggests that endogenization and selection of ERVs acting as restriction factors is a mechanism used by the host to fight retroviral infections.

## Introduction

An essential step in the replication cycle of retroviruses is the integration of their genome into the host genomic DNA. During evolution, exogenous retroviruses occasionally infected the germ cells of their hosts, resulting in stably integrated “endogenous” retroviruses (ERVs) that are passed to subsequent generations like any other host gene, following mendelian rules [1,2]. The continuous accumulation of new retroviral integrations over millions of years (a process also known as “endogenization”), which occurred by both reinfection and possibly intracellular retrotransposition, resulted in the genomes of all vertebrates being heavily colonized by ERVs (e.g., ERVs constitute ∼8% of the human genome) [3,4]. Most ERVs have accumulated genetic defects that render them unable to express infectious virus or proteins. However, some ERVs are transcriptionally active and have maintained intact open reading frames for some of their genes, raising the possibility that some of these elements may be beneficial to their hosts [5].

One of the possible explanations for the selection of some ERVs in vertebrates is their ability to provide protection against infection of related exogenous pathogenic retroviruses. ERVs can interfere with exogenous retroviruses by various mechanisms. For example, the expression of envelope (Env) glycoproteins by some ERVs in chicken and mice can block viral entry of exogenous retroviruses by receptor competition [6–8]. Expression of a truncated Gag from an ERV (Fv-1) in specific lines of mice confer protection against some strains of murine leukemia virus [9,10].

Sheep (Ovis aries) provide an exceedingly interesting animal model system to study retrovirus–host coevolution in nature. The history and timing of sheep domestication and breed selection is known and well documented by historical and archaeological records [11]. Jaagsiekte sheep retrovirus (JSRV) and enzootic nasal tumor virus (ENTV) are two related exogenous pathogenic retroviruses (of the genus *Betaretrovirus*) that cause naturally occurring neoplasms of the respiratory tract of sheep [12–14]. Interestingly, the sheep genome harbors many copies of ERVs highly related to JSRV/ENTV that are termed enJSRVs [14–17]. enJSRVs are abundantly expressed in the genital tract of the ewe, particularly in the endometrial lumenal and glandular epithelia of the uterus and in the trophectoderm of the placenta [14,18−20].

enJSRVs possess several biological features that support the idea of essentially a “symbiotic” relationship with their host. Recently, we demonstrated that enJSRVs are critical for reproduction of sheep, because inhibition of enJSRVs Env production in utero severely compromised trophectoderm growth and differentiation in the ovine placenta [21,22]. Similar biological roles in placental differentiation for different ERVs Env glycoproteins have been proposed in both humans and mice [23–28]. In addition, we found that some enJSRVs interfere with the replication of JSRV in vitro both by receptor competition and, most interestingly, at late stages of the replication cycle (a mechanism termed JLR, meaning JSRV late restriction) [29–31]. JLR is exerted by a defective transdominant enJSRV provirus, enJS56A1, whose Gag protein associates with and blocks intracellular trafficking of the JSRV Gag [30]. The major determinant of JLR is amino acid residue 21 in Gag that, in the exogenous JSRV/ENTV, is an arginine (R21) and in the transdominant enJS56A1 is a tryptophan (W21).

Our previous studies imply that sheep *Betaretroviruses* offer unique opportunities to study the coevolution of ERVs, exogenous retroviruses, and their hosts. Here, by characterizing the evolutionary history and molecular virology of enJSRVs we provide data supporting a model of continuous coadaptation between retroviruses and their hosts. In particular, these studies determined that: (i) two enJSRV loci that entered the host genome before and during speciation within the genus *Ovis*, acquired in two temporally distinct events, the W21 residue in Gag conferring a transdominant blocking phenotype; (ii) both these transdominant enJSRVs loci became fixed in the host genome before or around sheep domestication; (iii) the invasion of the sheep genome by ERVs of the JSRV/enJSRVs group is still in progress; and (iv) proviruses recently emerged that can escape the transdominant enJSRV loci. This study provides a unique outlook at the complex interplay between endogenous and exogenous retroviruses during host evolution.

## Results

### Isolation of enJSRV Proviruses with Either Intact or Defective Structure from the Sheep Genome

**Figure 1:**
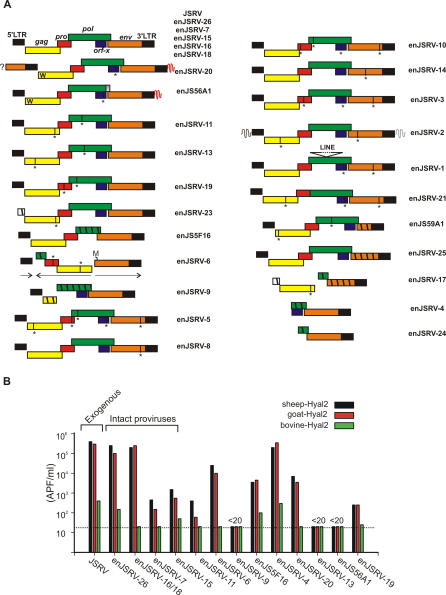
Genomic Organization of the enJSRV Proviruses and Cellular Receptor Usage (A) Genomic organization of the enJSRVs group. Five proviruses contained the typical intact genomic organization of the replication competent exogenous JSRV (top). The “W” in the *gag* reading frame of enJS56A1 and enJSRV-20 indicates the R21W substitution present in these two transdominant proviruses. Before the proximal LTR, enJSRV-20 contains a portion of an *env* gene indicated by the red box and a question mark. Stop codons are indicated by vertical lines and an asterisk (*). Large deletions in the proviruses are indicated by hatched boxes. The letter M in enJSRV-6 indicates the position of the first methionine (M) in *env* after the usual start codon present in the other enJSRV loci and the exogenous JSRV. enJSRV-6 contains a recombined structure with internal sequences present in the opposite direction compared to the 5′/3′ LTRs and the *env* gene (indicated by horizontal arrows). In all but two (enJSRV-2 and enJSRV-20) of the proviruses with both 5′ and 3′ LTR, a 6-bp duplication of the genomic DNA at the site of proviral integration was found that is typical of retroviruses. In contrast, enJSRV-2 has two different genomic sequences flanking the provirus, suggesting that this provirus is the likely product of recombination between two proviruses. enJSRV-20 contained a portion of *env* before the 5′ LTR, suggesting either an altered reversed transcription before integration or a recombination event. The 3′ flanking region of enJS56A1 and enJSRV-20 are essentially identical. enJSRV-1 presents a LINE element within the *pol* reading frame. (B) Receptor usage of exogenous and endogenous betaretroviruses of sheep. Viral entry assays were performed in NIH3T3 cells expressing sheep Hyal2 (ovine Hyal2), goat Hyal2 (goat Hyal2), or bovine Hyal2 (bovine Hyal2). Cells were transduced with retroviral vectors expressing alkaline phosphatase and pseudotyped with envelopes derived by the various enJSRVs indicated in the figure. Results are expressed as alkaline phosphatase foci per milliliter (APF/ml) and are indicated as <20 when the titer was less than 20 APF/ml.

We screened a sheep genomic bacterial artificial chromosome (BAC) library (CHORI-243), derived from DNA collected from a single Texel ram, and isolated, completely sequenced, and characterized 26 individual enJSRV proviruses including the previously cloned enJS56A1 and enJS5F16 (Figure 1A) [14]. One of the enJSRV proviruses (enJS59A1) that we identified previously [14] was not reisolated in this study, because our screening strategy employed probes in the *env* region that is deleted in enJS59A1.

Sequence analysis of the genomic sequences flanking the proviruses, including the six nucleotides duplicated upon proviral integration, served to unequivocally identify the various enJSRV loci. Proviruses cloned in this study were named enJSRV-1, −2, and so forth. Thus, the sheep genome contains at least 27 enJSRV proviruses. Previous studies estimated the presence of 15–20 enJSRV loci in the sheep genome by Southern blotting hybridization using *gag* and *env* probes [15].

We identified five proviruses (enJSRV-7, −15, −16, −18, and −26) with intact genomic organization and uninterrupted open reading frames for all the viral genes (*gag*, *pro*, *pol*, *orf-x*, and *env*) that is typical of replication competent JSRV/ENTV. Four of the five intact proviruses have identical 5′ and 3′ long terminal repeats (LTRs), which is suggestive of relatively recent integration or endogenization into the host germline (see below). Furthermore, two proviruses (enJSRV-16 and −18) are identical to each other at the nucleotide level along the entire genome, reinforcing the notion of recent integrations. Of the 27 enJSRV loci, 16 have an intact *env* open reading frame although two of these proviruses (enJSRV-4 and enJSRV-24) lack the 5′ LTR, *gag*, *pro*, and most of *pol*.

Interestingly, we identified another provirus (enJSRV-20) with a tryptophan residue in Gag position 21 (W21) conferring a defective and transdominant phenotype as with enJS56A1 [29,32]. enJSRV-20 had a portion of an *env* gene immediately before the proximal LTR and the 3′ genomic flanking sequence identical to the enJS56A1 homologous region. This suggests that enJSRV-20 was involved (or maybe derived in part) by processes of recombination/gene conversion with enJS56A1. However, enJS56A1 and enJSRV-20 are two distinct proviruses that can be distinguished by nucleotide sequence differences throughout the genome (23 nucleotides including a 2-bp deletion in *pol* present in enJS56A1 but not in enJSRV-20) and by the 5′ flanking region. Thus, there are at least two transdominant enJSRV proviruses in the sheep genome. Curiously, 19 of the 86 BAC clones that resulted positive for enJSRV sequences contained the enJS56A1 provirus, as judged by direct sequencing of the genomic region flanking the proximal LTR. All the other proviruses were present in one to five BAC clones with the exception of enJSRV-7 (present in nine BAC plasmids). The data suggest that the chromosomal location containing the enJS56A1 provirus may be duplicated in the sheep genome, considering that the BAC library used in this study has a 5× coverage of the sheep genome. However, we cannot rule out that the overrepresentation of some BAC clones is simply due to artifacts in the preparation or screening of the library.

enJSRVs have a very high degree of similarity with the exogenous sheep betaretroviruses JSRV and ENTV. For example, there is 85%–89% identity at the nucleotide level in *gag* and *env* between the infectious molecular clone JSRV_21_ [12] and the various enJSRV loci. However, all the enJSRV loci display the typical genetic “signatures” that differentiate them from the related exogenous pathogenic betaretroviruses. Major differences between enJSRVs and JSRV/ENTV are present in the U3 region of the LTR, in the variable region 1 (VR-1) and VR-2 in *gag*, and in *env* VR-3 as observed in the previously characterized enJSRV proviruses or PCR-derived enJSRV sequences from the sheep genome [14,33–35].

The JSRV/ENTV Env glycoprotein functions as a dominant oncogene in vitro and in vivo, and VR-3 is a major determinant of cell transformation [36–39]. None of the enJSRV loci isolated in this study presented VR-3 sequences similar to the exogenous JSRV/ENTV. Moreover, we were unable to induce foci of transformed cells in classical transformation assays of rodent fibroblasts transfected with expression plasmids of the various enJSRV loci that maintain an intact *env* open reading frame (unpublished data). These data suggest that retroviruses carrying the oncogenic JSRV/ENTV Env cannot be successfully selected as ERVs.

Using retrovirus vectors pseudotyped by the various enJSRVs Env glycoproteins in standard entry assays (Figure 1B), we confirmed that most enJSRV proviruses express a functional Env as determined by their ability to mediate virus entry using sheep hyaluronidase 2 (Hyal2), which can serve as a cellular receptor for the exogenous JSRV, ENTV, and the endogenous enJS5F16 Env [31,40–42]. Only vectors pseudotyped by enJSRV-26, enJSRV-16 (whose Env is identical to enJSRV-18), and enJSRV-4 Env showed titers similar to the vectors pseudotyped by the JSRV Env. The other enJSRV Env glycoproteins mediated entry less efficiently (approximately 100-fold) than the JSRV Env. All enJSRVs Env used goat Hyal2 as efficiently as ovine Hyal2, while bovine Hyal2 did not mediate entry very efficiently. enJSRV-9, enJSRV-13, and enJS56A1 were not able to mediate viral entry in 3T3 cells overexpressing sheep and goat Hyal2 or in other cell lines such as the ovine endometrial stromal cell line (oST) and goat embryo fibroblast cell line (TIGEF) (unpublished data). Thus, we conclude that the latter proviruses likely express defective Env proteins that do not efficiently mediate cell entry.

### Evolutionary History of enJSRVs

We next investigated the evolutionary history of enJSRVs in order to better understand the nature of the association of these viruses with their host.

The “age” of an ERV can be estimated by assessing the sequence divergence between the proximal and distal LTRs within a provirus, as it can be assumed that they were identical at the moment of proviral integration. The divergence accumulated between the LTRs over time can be used as a molecular clock (∼2.3–5 × 10^−9^ substitutions per site per year) [43]. However, LTR recombination and gene conversion occur frequently both within the same provirus and between different proviruses [44–46]. Consequently, this analysis can be used only as a general estimate of the age of a particular group of ERVs. Eight of 24 enJSRV proviruses possess identical LTRs, while the remaining proviruses display from one to eight nucleotide differences (0.2%–1.8%) (Table S1), resulting in an estimated time of integration of these ERVs from less than 450,000 y ago to around 8 million y ago (MYA). This period spans most of the evolutionary history of the subfamily of the Caprinae [47].

The time of integration of an ERV can be also estimated by determining whether phylogenetically related animal species share a particular provirus in the same genomic location, as this would imply an integration event before the split of those animal species during evolution. In this case, the calibration date relies on what is known for the evolutionary history of the animal species studied. Different studies estimate variable times of divergence among genera and/or species within ruminants and the Caprinae in particular, mainly because this subfamily had an extremely rapid expansion during some intervals in the Miocene and Pliocene [47]. Despite these limitations, we determined the distribution of each enJSRV provirus in domestic sheep (O. aries) and in other species within the subfamily Caprinae as this analysis can offer important data on virus-host coevolution. Samples tested included DNA collected from “wild sheep” of the *Ovis* genus (O. dalli, Dall sheep*; O. canadensis*, bighorn sheep; O. ammon, argali) and members of the genera *Hemitragus* (H. jenlahicus, Himalayan tahr), *Budorcas* (B. taxicolor, takin), and *Capra* (C. hircus, domestic goat*; C. falconeri*, markhor). Among the domestic sheep, we initially analyzed 20 sheep from ten different breeds (Dorset, Hampshire Down, Jacob, Merino, Rambouillet, Red Masai, Romanov, Southdown, Suffolk, and Texel).

**Figure 2:**
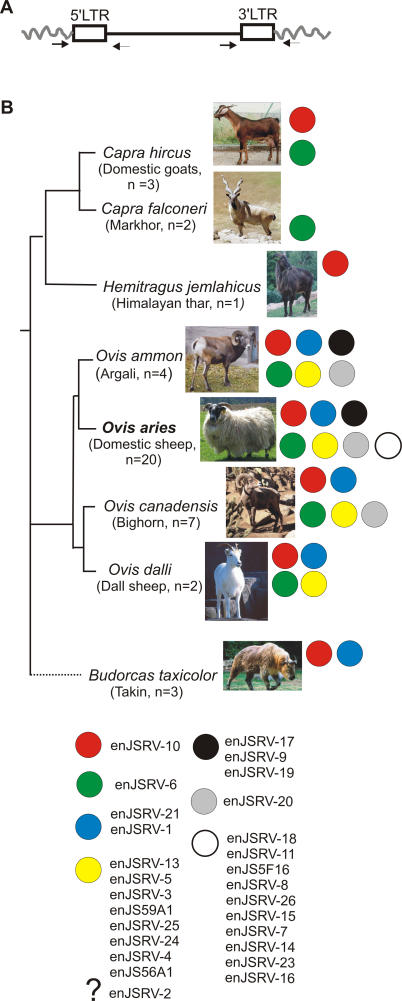
Distribution of enJSRVs within the Caprinae (A) Schematic representation of the PCRs used to specifically amplify each enJSRV provirus. (B) Simplified phylogenetic tree (branch length are not shown to scale) that shows representative species of the Caprinae subfamily used in this study. Common names and number of samples tested is indicated in parenthesis below the scientific names of each animal species. Colored circles beside the picture of each species symbolize the group of enJSRV loci indicated at the bottom of the figure that are present at least in some individuals of that particular species. The question mark indicates that the PCR for enJSRV-2 could not be optimized and has not been used in this study. Please note that the position of the genus *Budorcas* in the phylogenesis of the Caprinae is not well understood, thus it is schematically represented with a broken line [45]. Images of the various animal species were kindly provided by Brent Huffman (http://www.ultimateungulate.com/).

As schematically illustrated in Figure 2A, we analyzed each enJSRV locus using two primer sets that amplified, respectively, the junction between the provirus and the genomic flanking sequence at the 5′ or 3′ end of the provirus. As depicted in Figure 2B, two proviruses, enJSRV-10 and enJSRV-6, were shared across various genera, suggesting that they integrated before the split of the genus *Ovis* (sheep-like species) from the genus *Capra* (goat-like species) that is estimated to have occurred between 5 and 7 MYA [47–49]. PCR amplification of enJSRV-6 (or enJSRV-10) was used to control for the quality of the DNA samples in all experiments.

Ten proviruses (including enJS56A1) were common between the domestic sheep and members of the genus *Ovis*, including bighorn sheep (O. canadensis), Dall sheep (O. dalli) and argali (O. ammon). The origin of the *Ovis* genus is estimated to have occurred approximately 3 MYA [47,50], with the early *Ovis* prototypes giving origin to the North American bighorn sheep and Dall sheep. Three proviruses were in common only between the argali and the domestic sheep and were not present in the bighorn and Dall sheep. enJSRV-20 was shared among bighorn sheep, argali, and domestic sheep. The argali diverged from the domestic sheep between 0.4 and 1.3 MYA [50]. Ten proviruses were present only in the domestic sheep. Most interestingly, we detected insertionally polymorphic proviruses. Seven of ten proviruses present in the domestic sheep were present only in some of the sheep tested, while one provirus (enJSRV-26) was not detected in any of the samples (see below).

These results, combined with the present knowledge of ruminant evolution, suggest that enJSRV integration began 5–7 MYA before the split of the genus *Ovis* from the genus *Capra* and continued after sheep domestication (∼ 9,000 y ago) [11]. PCR analyses using primers within conserved regions in the enJSRVs *gag* or *env* did not identify enJSRVs in the more distantly related Bos taurus (domestic cow) or Odocoileus hemionous (Black-tail deer) (unpublished data).

### Selection of enJS56A1 and enJSRV-20 with Transdominant Genotypes in Ovis aries

At least two proviruses in the sheep genome, enJS56A1 and enJSRV-20, possess a tryptophan residue in Gag position 21 (W21) that confers a defective and transdominant phenotype [29,32]. Indeed, we previously showed that residue R21 in Gag is required for JSRV exit and the JSRV R21W single mutant is defective and transdominant [29,32]. Therefore, we hypothesized that enJS56A1 (and possibly enJSRV-20) originally possessed residue R21 in Gag, rather than W21, when first integrated into the host germline. In this model, the transdominant W21 Gag residue would subsequently be acquired and selected due to the ability of the transdominant proviruses to interfere with related replication competent retroviruses and/or to reduce fitness of other enJSRVs loci.

**Figure 3:**
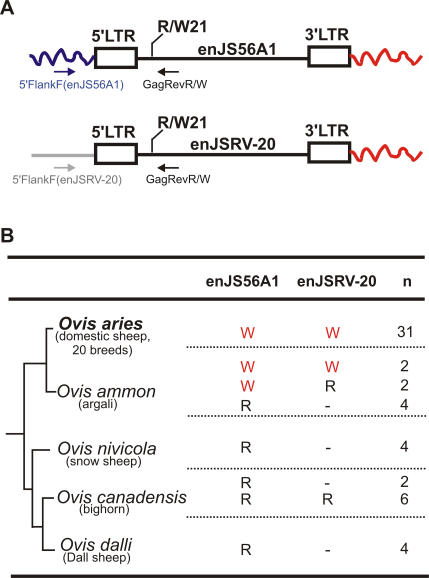
Fixation of R21W Substitution in enJS56A1 and enJSRV-20 Gag in the Domestic Sheep (A) Schematic representation of the PCRs used to specifically amplify the 5′ LTR and the proximal region of *gag* of enJS56A1 or enJSRV-20. (B) Schematic representation of the presence or absence of the transdominant genotypes of enJS56A1 and/or enJSRV-20 in *Ovis* species. The phylogenetic tree indicates only relationships and is not proportional to time.

To experimentally address this model, we sequenced the beginning of the *gag* gene in proviruses enJS56A1 and enJSRV-20 amplified from different individuals of different Caprinae species. The data obtained support the idea of relatively recent selection of the W21 Gag residue in both enJS56A1 and enJSRV-20 (Figure 3). Both proviruses exhibited the W21 Gag residues in 31 domestic sheep (O. aries) sampled from 20 breeds originating from different geographical locations. In contrast, we found variability in the eight argali (O. ammon) tested where only four of eight animals possessed both enJS56A1 and enJSRV-20. Two of those argali harbored both enJS56A1 and enJSRV-20 with the W21 residue in Gag, whereas the other two argali displayed residue W21 in Gag of enJS56A1 and R21 in enJSRV-20. The remaining four argali did not harbor enJSRV-20 and contained the Gag R21 residue in enJS56A1. enJSRV-20 was not detected in the snow sheep (O. nivicola), Dall sheep (O. dalli) and two bighorn sheep (O. canadensis), while enJS56A1 possessed the R21 Gag residue in all these animals (*n* = 10). Most interestingly, six bighorn sheep displayed the R21 residue in Gag in both enJS56A1 and enJSRV-20. These data strongly argue that both enJS56A1 and enJSRV-20 possessed the wild type R21 residue in Gag when they originally integrated into the host germline. Subsequently, two temporally distinct events resulted in the presence of two transdominant proviruses with the W21 Gag residue, which eventually became fixed in the domestic sheep (see also Discussion).

### Recently Integrated enJSRV Proviruses

The presence of insertionally polymorphic enJSRV loci found in none or a few of the sheep tested suggests that these proviruses integrated relatively recently and presumably around or after domestication.

**Figure 4:**
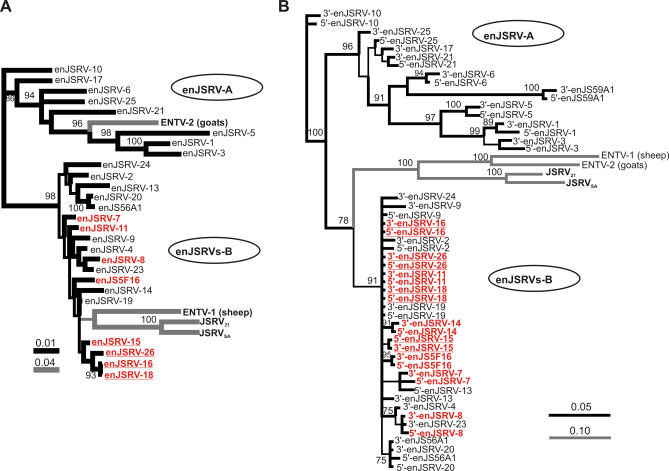
Phylogenetic Relationships among Endogenous and Exogenous Sheep Betaretroviruses (A) Phylogenetic tree based on the sequences of the full provirus (excluding LTRs). (B) Phylogenetic tree based only on LTR sequences. Genealogies shown represent Bayesian 50% majority rule consensus trees and were rooted using isolate enJSRV-10, which is shared among *Ovis* and Capra spp. Clades with posterior probability values at least 0.95 are indicated by thick branches. Bootstrap scores 70% or above from ML analysis (based on 200 replicates) are shown above branches. For the larger LTR dataset, 1,000 replicates were analyzed using the neighbor-joining method with distances calculated from the ML model. Branches in grey are shown at smaller scales, which allowed for easier graphical representation of both fast-evolving exogenous and slow-evolving endogenous forms in the same tree figure. Two well-supported clades are visible in both trees. Provirus names in red indicate the insertionally polymorphic loci, while those underlined are the enJSRVs that show an intact genomic organization with complete uninterrupted open reading frames.

Phylogenetic analysis of enJSRVs and the exogenous JSRV/ENTV divided these retroviruses into two clades, which can be referred to as enJSRV-A and enJSRV-B (Figure 4) [14]. All of the insertionally polymorphic enJSRVs group within the enJSRV clade B while most of the oldest proviruses group within enJSRV clade A. Interestingly, the exogenous viruses of sheep (JSRV and ENTV-1) group with enJSRVs of clade B, whereas ENTV-2, which was isolated from goats, groups with enJSRVs clade A in the phylogenetic trees that were constructed using the entire proviral genome (without the LTRs) (Figure 4A) or *env* (Figure S1). In the phylogenetic trees constructed using the LTR sequences, all the exogenous viruses cluster in an independent clade (Figure 4B). The transdominant enJS56A1 and enJSRV-20 cluster together in all the phylogenetic analyses performed.

In order to better characterize the distribution of the insertionally polymorphic enJSRV loci we extended our analysis using 330 samples collected from 27 different sheep breeds (Table 1). As mentioned above, the CHORI-243 BAC library was derived from a single Texel ram. The Texel breed was developed approximately 200 years ago on the island of Texel in the Netherlands from a local breed improved by British breeds that included Leicester, Lincoln, Wensleydale, Hampshire Down, and others. Our samples include British breeds used in the development of the Texel. Five proviruses, enJSRV-7, −8, −11, −18, and enJS5F16 were present in 30%–100% of the Texel sheep and in approximately 12%–80% of the remaining sheep from other breeds. Two other proviruses, enJSRV-15 and enJSRV-16, were detected with a frequency of around 30% in Texel sheep, but only in 1%–4% of other breeds.

**Table 1:**
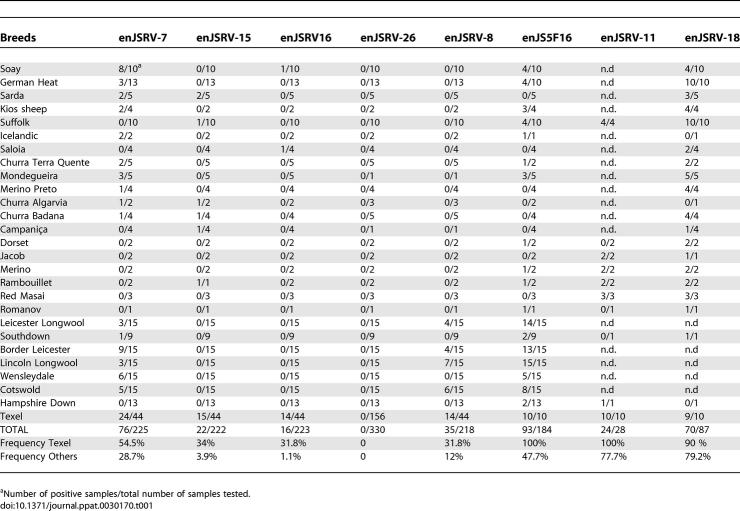
Relative Frequency of Insertionally Polymorphic Proviruses in Sheep

Full length endogenous proviruses can occasionally originate “solo LTRs” by homologous recombination between their 5′ and 3′ LTRs. Consequently, a negative result in our PCR assays could be also due to “solo LTRs” rather than from the complete absence of a provirus from that particular genomic location. We ruled out the possibility that our negative samples contained solo LTRs by performing three different PCR tests using (i) both PCR primers in the flanking genomic regions (i.e., amplifying the empty genomic insertion site) and (ii and iii) primer pairs with, respectively, one primer in the LTR and one in the 5′ or 3′ genomic flanking region (Figure S2). In all cases we amplified the empty genomic insertion sites while we did not obtain any evidence of solo LTRs. Samples screened included all the samples determined negative for enJSRV-11 and enJSRV-18 and approximately one sample per breed for the other insertionally polymorphic proviruses. A minimum of 20 samples for each provirus were tested.

**Figure 5:**
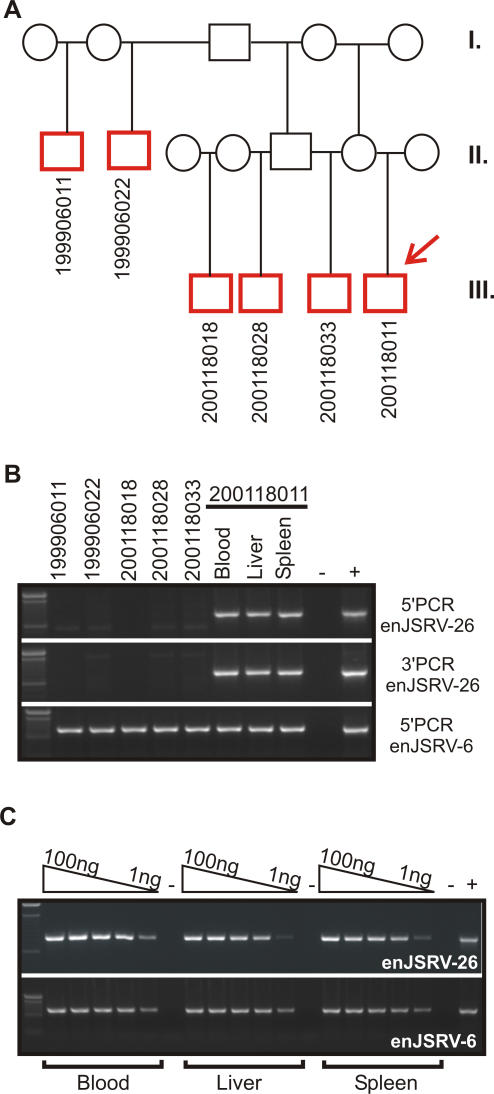
enJSRV-26 Is an Endogenous Retrovirus (A) Genealogical tree illustrating the relationship between ram #200118011 (indicated by an arrow) and its relatives, whose DNA samples were analyzed in this study. The BAC library used in this study was derived from ram #200118011. (B) Detection of enJSRV-26 by amplification of the 5′ and 3′ provirus-flanking genomic sequences. enJSRV-26 was detected in blood, liver, and spleen DNA from ram #200118011. Five relatives of ram #200118011 (three half siblings and two sons of the ram grandsire) did not harbor enJSRV-26. The PCR for enJSRV-6 was used as a control for genomic DNA quality. (C) enJSRV-26 was present at relatively the same frequency in blood and tissues of ram #200118011.

Remarkably, we did not detect enJSRV-26 in any of the sheep tested (*n* = 330), including 156 Texel sheep sampled from different flocks in the UK, Denmark, and US, and 82 sheep from British breeds used to develop the Texel breed. Thus, enJSRV-26 was present only in the original CHORI-243 BAC library that was made using DNA from a single Texel ram. The lack of enJSRV-26 in the large number of samples tested could be due to this provirus being (i) an ERV very recently integrated in this breed or (ii) a previously unrecognized exogenous retrovirus infecting the ram whose blood was used to construct BAC library CHORI-243. Fortunately, this library was constructed using DNA from a known source, ram #200118011 from the US Meat Animal Research Center (USMARC) in Nebraska. Blood and tissues (liver and spleen) of this ram along with the blood of three half-siblings (animals #200118018, #200118028, and #200118033) and two sons of its grandsire (rams #199906011 and #199906022) were available for our studies (Figure 5A).

We detected enJSRV-26 in the blood and all other tissues from ram #200118011, but not in the DNA from its five relatives (Figure 5B). Importantly, enJSRV-26 was present with the same frequency in blood and tissue DNA of ram #200118011 (Figure 5C); therefore enJSRV-26 is indeed an extremely rare ERV (possibly present in a single animal) that likely integrated in the sheep germline less than 200 years ago after development of the Texel breed.

### enJSRV-26 Produces Viral Particles In Vitro and Escapes JLR Restriction

It is possible that at least some of the insertionally polymorphic proviruses represent exogenous retroviruses that have been circulating (or still circulate) and spreading horizontally in the sheep population. This finding raises the question of how these viruses might be transmitted in the presence of transdominant ERVs.

**Figure 6:**
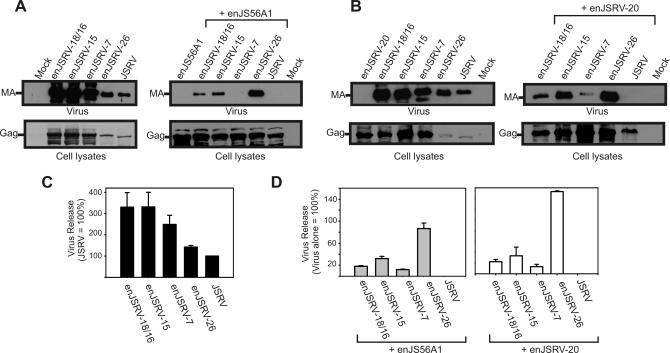
enJSRV-26 Escapes Restriction by enJS56A1 and enJSRV-20 (A and B) 50 μg of cell lysates (bottom) and virus pellets from supernatants (top) of cells transfected with plasmids expressing the indicated viruses were analyzed 48 h post-transfection by SDS-PAGE and Western blotting employing an antiserum against the JSRV p23 (Matrix, MA). (C and D) viral pellets were quantified by scanning Western blot membranes and measuring chemifluorescence in a Molecular Dynamics Storm 840 imaging system using ImageQuant TL software. Results are presented as the means (± standard error) obtained in respectively six (C) and three (D) independent experiments. In (C) values are normalized with JSRV (designated as 100% viral release) while in the cotransfection assays 100% is taken as the value of each individual virus expressed by itself without enJS56A1 or enJSRV-20.

Due to the lack of an in vitro tissue culture system for the propagation of JSRV, we determined the capacity of the intact enJSRV proviruses to produce viral particles in vitro by expressing them in transient transfection assays. We constructed expression vectors for each intact enJSRV provirus by replacing the proximal U3 sequence in the LTR, which contains the retroviral promoter and enhancers, with the cytomegalovirus (CMV) immediate early promoter. We have used this system to produce exogenous JSRV virions that are infectious and oncogenic in vivo [12]. We derived vectors for only four of the five proviruses, because enJSRV-16 and enJSRV-18 are 100% identical. All enJSRV proviruses expressed abundant viral particles in the supernatant of transfected cells (Figure 6). Despite the constructs containing the same CMV promoter, enJSRV-7, −15, and −16/18 reproducibly released ∼3-fold more viral particles in the supernatant of transfected cells than JSRV or enJSRV-26 (Figure 6A and 6C).

Next, we investigated whether the transdominant proviruses enJS56A1 and enJSRV-20 could block the release of the intact enJSRV proviruses as efficiently as the exogenous JSRV. Standard interference assays were conducted in which cells were transfected with the expression plasmids for the intact proviruses either by themselves or with the transdominant proviruses. Cotransfection of enJS56A1 and enJSRV-20 caused a sizable decrease (∼70%–85%) in release of viral particles from enJSRV-7, −15, and −16/18 and completely inhibited release of viral particles from JSRV. However, neither enJS56A1 nor enJSRV-20 blocked enJSRV-26 viral exit. We reproducibly detected more viral particles in the supernatants of cells cotransfected by enJSRV-26 and enJSRV-20 than in cells transfected with enJSRV-26 alone. These data suggest that enJSRV-26 could also rescue enJSRV-20.

Consequently, the most recently integrated enJSRV provirus is the only one, among the JSRV/enJSRVs group of viruses, which is able to escape JLR.

## Discussion

ERVs have been regarded over the years as either nonfunctional “junk DNA” or important contributors to fundamental biological functions including mammalian placentation [21–26,51] and maintenance of genomic plasticity [44]. Some ERVs have been found to interfere with the replication of their exogenous counterparts [1,2]. However, it has been difficult to assess whether retrovirus endogenization has indeed helped the host during evolution to cope with pathogenic retrovirus infections. In this study, we investigated the evolutionary interplay between endogenous retroviruses, exogenous retroviruses, and their hosts, using the sheep as a model system. The data presented here revealed sequential, coadaptive genetic changes between endogenous and exogenous betaretroviruses of sheep that alternatively favored either the host or the virus. The identification of an ERV (enJSRV-26), which likely integrated in the host genome in the last 200 y and escapes transdominant enJSRVs, suggests that the evolutionary antagonism between endogenous and exogenous betaretroviruses in sheep is a continuous process that has not reached equilibrium. The data obtained strongly suggest that ERVs have been used by the host to fight exogenous pathogenic retroviral infections.

**Figure 7:**
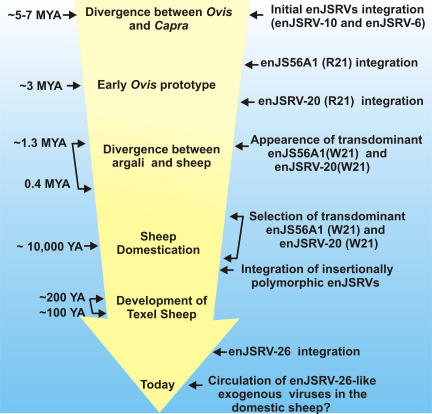
Dates and Events Associating the Evolutionary History of enJSRVs and Their Host Schematic diagram illustrating key events of the evolutionary history of enJSRVs described in this study in association with estimated dates during the evolution of the domestic sheep and the Caprinae.

enJSRVs have been integrating into the host genome during a period that spans most of the evolutionary history of the Caprinae (5–7 MYA). By tracking the evolutionary history of these proviruses we unveiled fascinating events underscoring the host-virus “struggle” over several million years (Figure 7). The transdominant enJS56A1 and enJSRV-20 possessed the “wild type” R21 Gag residue when they first entered the genome of the host. The transdominant W21 Gag residue rose to fixation in enJS56A1 and enJSRV-20 in two temporally distinct events around or before sheep domestication. Given the high degree of similarity between enJSRV-20 and enJS56A1 and the identity of their 3′ flanking region, it appears most likely that a process of gene conversion (rather than independent mutations) conferred the transdominant Gag to enJSRV-20. Indeed enJSRV-20 might be the result of various processes involving recombination between other proviruses and enJS56A1. One of these processes resulted in a portion of an *env* gene upstream the enJSRV-20 proximal LTR. All the PCR assays used in this study to amplify the 5′ LTR and the *gag* gene of enJSRV-20 employed a forward PCR primer overlapping *env* and a reverse primer in *gag*. Consequently, we always amplified the enJSRV-20 locus possessing a portion of *env* flanking the proximal LTR even in those bighorn sheep and argali with the Gag R21 residue in this provirus. Thus, the possible recombination event resulting in part of an *env* gene flanking the proximal LTR of enJSRV-20 likely preceded the appearance of the transdominant W21 Gag residue in this provirus. Other scenarios are also feasible. For example, the presence of the R21 Gag residue in the enJSRV-20 provirus possessed by some argali and bighorn sheep may derive from reverted mutations of the transdominant W21 Gag residue. Whatever the case, our data suggest that the fixation of transdominant proviruses happened at least twice in the *Ovis* genus.

Overall, the data obtained are highly suggestive of positive selection of transdominant proviruses, although we cannot rule out the possibility that a genetic bottleneck during sheep domestication also contributed to this process. Sheep domestication occurred ∼9,000 years ago in the Middle East during the Neolithic agricultural revolution [52–54]. Sheep, like other domestic animals, have different maternal haplotypes that are believed to be the result of independent centers of domestication. There are at least five highly divergent mtDNA haplogroups in sheep worldwide (the most common A, B, C, and the rarer D and E) [55,56]. Most studies date the separation of the two most frequent haplogroups (A and B) well before domestication per se occurred [55]. Therefore, the most likely explanation for this high level of molecular divergence is that genetically differentiated subspecies of the ancestral wild sheep were domesticated in different regions of Near East and Asia. Currently, the three most common haplogroups (A, B, and C) have a widespread geographical distribution, coexisting in some cases in the same breed. Among the 31 samples from domestic sheep that we tested for the presence of the transdominant W21 residue in enJS56A1 and enJSRV-20, we had six samples from Portuguese breeds (Saloia, Churra Terra Quente, Churra Algarvia, and Churra Badana) where the maternal haplotypes had been determined in a previous study and represented the most common haplotypes A, B, and C [57]. Thus, sheep from diverse mtDNA haplotypes contain the transdominant enJSRV-20 and enJS56A1. Under this scenario, we favor the hypothesis that the selection of the W21 Gag residue in enJS56A1/enJSRV-20 in all domestic sheep was due to positive selection rather than a random founder effect. Given the multiple origins of domestication in sheep, it is feasible that the transdominant enJS56A1 and enJSRV-20 assisted the host in coping with the likely increased exposure to exogenous retroviruses that resulted from the concentration of animals into herds, which increased population density, thus favoring virus transmission. It is also possible that only wild individuals with the transdominant proviruses were selected and domesticated in different locations at different times. The transdominant proviruses might have been selected to defend the host against related ERVs already colonizing the host genome rather than against pathogenic exogenous retroviruses. In either scenario, the R21W mutation in Gag would increase fitness of their host and furnish an adaptive advantage.

Interestingly, we found eight enJSRV proviruses that were present only in a proportion of tested sheep. Five of the insertionally polymorphic loci have an intact genome, a functional Env, and were able to produce viral particles in vitro. Three of these proviruses were present in the sheep population at a very low frequency. In particular, enJSRV-26 was present only in a single Texel ram that was fortuitously the donor of DNA used for construction of the BAC library screened in this study.

The Texel breed of sheep was developed on an island off the coast of The Netherlands (Island of Texel) at the end of the 19th century by crossing local sheep with British breeds including the Leicester, Lincoln, and Hampshire Down. In 1909, the Texel sheep herdbook was created, the crossing was stopped, and the breed was established. enJSRV-26 was not detected in 330 sheep from 27 different breeds including 156 Texel originating from different geographical locations (UK, US, and Denmark) and 82 sheep from the British breeds that contributed to development of the Texel. Interestingly, enJSRV-26 escapes JLR restriction induced by enJS56A1 and enJSRV-20. Collectively, these data suggest that enJSRV-26 integrated very recently in the sheep genome, probably less than 200 y ago, and may even be a unique integration event in a single animal. The most likely scenario is that an exogenous retrovirus closely related to enJSRV-26 is circulating within the sheep population with an undetermined (if any) pathogenic effect. The presence of intact enJSRV loci in the sheep genome, which have a high degree of similarity to the pathogenic JSRV/ENTV but lack their oncogenic potential, strongly suggest the existence of enJSRV-like exogenous retroviruses. We showed that the enJSRVs Env glycoproteins, unlike the homologous JSRV/ENTV, do not function as dominant oncoproteins. Most likely the oncogenic JSRV/ENTV are not suitable to be selected as ERVs, because they should perturb cell physiology.

The high degree of similarity of enJSRV-26 with the other enJSRVs make its detection as a potential exogenous virus complicated, but it will be interesting to determine whether this virus could be associated with any disease process. JLR escape would provide an obvious evolutionary advantage to an enJSRV-26-like exogenous virus that may allow it to persist within the sheep population. In contrast, the exogenous JSRV lacks such an advantage but acquired a markedly different tropism from the enJSRVs due to major differences within their LTRs [58]. JSRV is highly expressed in the tumor cells in the lungs and its LTRs are activated by lung-specific transcription factors while the enJSRVs LTR respond to progesterone in vivo and in vitro and are expressed in the genital tract [19,58–60]. Therefore, we hypothesize that JSRV escapes enJSRVs-induced block by being able to replicate in tissues where enJSRVs are not expressed or are expressed at low abundance [14].

enJSRV-26 could also result from intracellular retrotransposition, possibly resulting by recombination of different proviruses. In any case, enJSRV-26 is one of the “youngest” ERVs cloned to date, reinforcing the idea that ERVs do not derive only from integration of exogenous retroviruses that occurred in the distant past.

ERVs have been classified as “ancient” or “modern” [61,62], depending on whether integration occurred before or after speciation. However, koalas with new germline integrations by ERVs were discovered recently [63,64]. Older studies found that particular inbred mouse strains could acquire new ERVs integrations in the germline [65,66]. Thus, there may be the need to define also “contemporary” ERVs that are still integrating in the germline of the host with potentially either beneficial or adverse effects. We predict that the increased availability of genomic tools will favor the discoveries of rare, recently integrated ERVs.

The presence of intact ERVs in the sheep is another example, beside porcine ERVs, of the attention that must be given to retroviruses when taking into consideration xenotransplantation [67,68]. The “sheep-human chimera” is a very promising experimental platform based on the transplantation of human embryonic stem cells in fetal sheep [69–72]. This model offers the theoretical possibility to develop sheep bearing organs with “human” characteristics available for xenotransplantation. In addition to the various ethical considerations, the presence in some sheep of intact enJSRV proviruses will have to be considered when devising strategies for the development of these chimeras.

In conclusion, this study has provided substantial novel evidence for the coevolution of ERVs, exogenous retroviruses, and their natural host. Endogenization and selection of ERVs acting as restriction factors for related viruses around or before sheep domestication and the recent appearance of proviruses escaping this block highlight the fact that biological interactions between ERVs and their host is, in some cases, an ongoing dynamic process and reinforces the hypothesis that ERVs played significant evolutionary roles in host defense.

## Materials and Methods

### enJSRVs cloning strategy.

The CHORI-243 sheep BAC library was constructed by BACPAC Resources at the Children's Hospital Oakland Research Institute (Cleveland, OH) following established procedures [73]. The library has a 182 (± 40) kb average insert size with individual clones arrayed on high-density hybridization filters. A segment of the library corresponding to a 5× genome coverage was custom screened by BACPAC Resources using hybridization with two probes (5′-CGGCTTTTTGGGCATACATTCCTG-3′ and 5′-TGAATCATAGGCGGATCAGGAATG-3′) corresponding to a highly conserved region in *env* of the known endogenous and exogenous sheep betaretroviruses. This screen identified 223 clones, and 182 of those clones were confirmed to contain enJSRV sequences by PCR employing primers pairs that amplify a region in *env* outside the one covered by the probe used in hybridization (5′-CAACGCATTAATACGGCTCTT-3′ and 5′-AATTAGCATGGCATTGAATTTT-3′). A total of 86 of the 182 BAC clones were further analyzed by sequencing. Redundant BAC clones were discarded by sequencing the 5′ and 3′ proviral insertion sites in each clone using primers in the untranslated *gag* region and at the end of the *env* gene. A total of 26 unique clones were identified, including two BAC clones containing the previously characterized enJS56A1 and enJS5F16 proviruses [14]. Southern blots were performed using LTR, *env*, and *gag* probes, which established that only one provirus was present in each BAC clone used to obtain the sequences of the various enJSRV loci (unpublished data). Both strands of individual enJSRV proviral clones were completely sequenced using BAC DNA as template. Occasionally, sequencing of PCR products obtained from the various BAC clones was used to confirm specific problematic sequences.

### enJSRVs nomenclature.

The previously characterized enJSRV loci were designated as enJS5F16, enJS56A1, and enJS59A1 [14]. For simplicity, the new enJSRV proviruses are designated with a numbering system (i.e., enJSRV-1, enJSRV-2, etc.). This system should allow an easier transition into a more uniformed nomenclature for all ERVs that is currently under development (J. P. Stoye, personal communication).

### Plasmids.

The intact proviruses enJSRV-7, enJSRV-15, enJSRV-18, and enJSRV-26 were subcloned by PCR into pBlueScript (Stratagene) and termed, respectively, pCMV5-enJS7, pCMV5-enJS15, pCMV5-enJS18, and pCMV5-enJS26. The U3 of the proximal LTR was replaced with the CMV immediate early promoter as previously described for the expression vector of the molecular clone JSRV_21_ [12] and for enJS56A1 [14]. All plasmids were completely sequenced in both directions to rule out PCR errors during the subcloning procedure.

Expression plasmids for the JSRV Env have been described previously [74]. Expression plasmids for the various enJSRV Env were derived from pCMV3JS21ΔGP by replacing the JSRV_21_ *env* with the homologous region of most of the loci that presented an intact *env* open reading frame and 5′ LTR (i.e. enJSRV-4, −6, −7, −9, −11, −13, −15, −16, −19, −20, −26, enJS5F16, and enJS56A1). The resulting plasmids were termed pCMVenJS4ΔGP, pCMVenJS6ΔGP, etc.

### enJSRV-specific PCRs.

The genomic DNA samples used in this study are described in the Results section. Specific amplification of each enJSRV provirus was obtained by two sets of PCR reactions (5′- and 3′- PCR) performed using 10–100 ng of genomic DNA and the HotStar Taq DNA polymerase system using standard amplification cycles and annealing temperatures between 57 °C and 59 °C as recommended by the manufacturer (Qiagen). PCR primers in the 5′-PCR were designed using a forward oligonucleotide primer (5′FlankF) complementary to the genomic flanking sequence adjacent to the 5′ LTR of each specific provirus and a reverse oligonucleotide primer (ProvR) complementary to the untranslated *gag* region or to the 5′ end of the viral genome in the deleted proviruses (i.e., enJSRV-4 and enJSRV-24). In order to sequence the proximal region of *gag* in enJS56A1 and enJSRV-20 we used the 5′FlankF primer with the reverse primer GagRevR/W (5′-ACTGTACCTTCTCTGGGGAACC-3′) as indicated in Figure 3A. The 3′-PCR used a forward oligonucleotide primer designed at the end of the *env* region (ProvF) and a reverse primer complementary to the genomic flanking genome adjacent to the 3′ LTR (3′FlankR). The quality of the DNA preparation was assessed in all samples by PCR amplification using primer pairs that amplify the insertion sites and LTR of enJSRV-6 or enJSRV-10 that, one or the other, are present in all the studied species. Each sample was scored as having a particular provirus when both the 5′ and 3′ PCR reactions resulted positive. The presence of solo LTRs was ruled out in a subset of samples for the insertionally polymorphic proviruses using three distinct PCR assays specifically designed to amplify solo LTRs and the empty genomic DNA around the proviral integration site. The strategy and complete list of primers used in the various PCR assays described above are shown in Figure S2.

### Phylogenetic analysis.

We estimated viral phylogenies using maximum likelihood (ML) and Bayesian methods. Because some enJSRV loci had lost substantial parts of their genome, phylogenetic analyses could not be carried out for a single alignment. In total, four datasets were assembled corresponding to: (i) the complete proviruses (without LTRs); (ii) LTRs (5′ and 3′); (iii) *pol*; and (iv) *env*. Alignments were obtained using the Clustal W method [75] as implemented in the program MegAlign (Lasergene software, DNASTAR). For each dataset, an appropriate model of evolution was selected from the suite of models included in Modeltest [76] using AIC (Akaike's information criterion) and model averaging [77]. ML phylogenies were found under heuristic search algorithms using TBR (tree bisection and reconnection) in Paup* v4.0b10 [78]. Clade support was evaluated based on 200 bootstrap replicates using the same search algorithm, except for the larger LTR data set in which 1,000 replicates were analyzed using the neighbor-joining method with distances calculated from the ML model. Bayesian estimates of phylogenies were obtained in MrBayes v3.1.2 [79]. Maintaining the general form of the previously selected substitution model (e.g., GTR + G), two independent chains were run for 5,000,000 generations each, of which 1,000,000 were removed as burn-in. Parameters and trees were sampled every 1,000 steps. This length was sufficient in all cases to reach convergence as indicated by split frequencies < 0.01.

### Cell culture, transfections, and viral preparations.

293T cells were cultured in Dulbecco's modified Eagle medium supplemented with 10% fetal bovine serum at 37 °C under 5% CO_2_ and 95% humidity. Virus was produced by transient transfection of 293T cells with the appropriate plasmids using linear polyethylenimine (Polysciences) as previously described [80]. Cell supernatants were collected at 24 and 48 h post-transfection, and viral particles were concentrated by ultracentrifugation as previously described [12]. For analysis of intracellular Gag, cells were lysed by standard techniques as described previously [30].

NIH-3T3 cells were grown in Dulbecco's modified Eagle medium supplemented with 10% calf serum. NIH-3T3 stably expressing Hyal2 from either sheep (sheep-Hyal2), goat (goat-Hyal2), or bovine (bovine-Hyal2) were produced by transduction with a standard retroviral vector expressing sheep, goat, or bovine Hyal2 followed by G418 selection (500 μg/ml). Both the ovine endometrial stromal cell line (oST) and the large TIGEF (T-antigen immortalized goat embryonic fibroblast) cell line were previously described [81,82].

### Entry assays.

The ability of the enJSRV Env proteins to mediate cell entry was assessed by standard entry assays using murine leukemia virus-based vectors. 293-GP-AP, a packaging cell line expressing murine leukemia virus Gag and Pol, and a standard retroviral vector expressing alkaline phosphatase has been described before [83]. 293-GP-AP cells were transfected with the expression plasmids for the various enJSRV Env or with plasmids expressing the exogenous JSRV Env as control. Supernatants were collected and stored at −70 °C. Subsequently, naïve NIH-3T3 and NIH-3T3 expressing either ovine (sheep-Hyal2), goat (goat-Hyal2), or bovine Hyal2 (bovine-Hyal2) were exposed to 10-fold serial dilutions of vector supernatants. Cells were fixed at 2 d postinfection and stained for alkaline phosphatase-positive foci. Viral titer is expressed as alkaline phosphatase foci per milliliter (APF/ml). Experiments were performed at least two times with two replicates tested for each dilution.

### Western blot analysis.

SDS-PAGE and Western blotting were performed on concentrated viral particles and cell lysates (50 μg of protein extracts) using methods described previously [12,30]. Gag proteins were analyzed with rabbit polyclonal sera against JSRV major capsid protein (CA) or MA (p23) [29,32] and an anti-rabbit peroxidase-conjugated antibody (Amersham) and detected by chemiluminescence using ECL Plus (Amersham). Western blots were quantified by scanning membranes and measuring chemifluorescence in a Molecular Dynamics Storm 840 imaging system using ImageQuant TL software (Molecular Dynamics). Experiments (from transfections to Western blotting) were performed independently at least three times and are presented as the mean value for each sample (± standard error).

## Supporting Information

Figure S1 Phylogenetic Relationships among Endogenous and Exogenous Sheep Betaretroviruses Phylogenetic tree based on the sequences of the *env* gene. The tree was constructed as indicated in the legend of Figure 4 and in Materials and Methods.
(80 KB PDF)

Figure S2 Strategy and Oligonucleotide Primers of the Various PCR Assays Used in This Study (33 KB PDF)

Table S1 Estimated Integration Time for Each enJSRV Provirus Based on the Differences between the 5′ and 3′ LTR (51 KB DOC)

### Accession Numbers

The sequences of the new enJSRV loci described in this paper are available in GenBank (http://www.ncbi.nlm.nih.gov/); accession numbers are EF680296 to EF680319.

## Abbreviations

BAC: bacterial artificial chromosome
CMV: cytomegalovirus
ENTV: enzootic nasal tumor virus
ERV: endogenous retroviruses
JLR: JSRV late restriction
JSRV: Jaagsiekte sheep retrovirus
LTR: long terminal repeat
MYA: million years ago
VR: variable region
