# Supplementary material for: A Paradigm for Virus–Host Coevolution: Sequential Counter-Adaptations between Endogenous and Exogenous Retroviruses
Source: PLoS Pathog. 2007 Nov 9;3(11):e170. doi: 10.1371/journal.ppat.0030170 (PMC2065879; doi:10.1371/journal.ppat.0030170)
Supplement: Supplementary file 1 [file ppat.0030170.sg001.pdf]

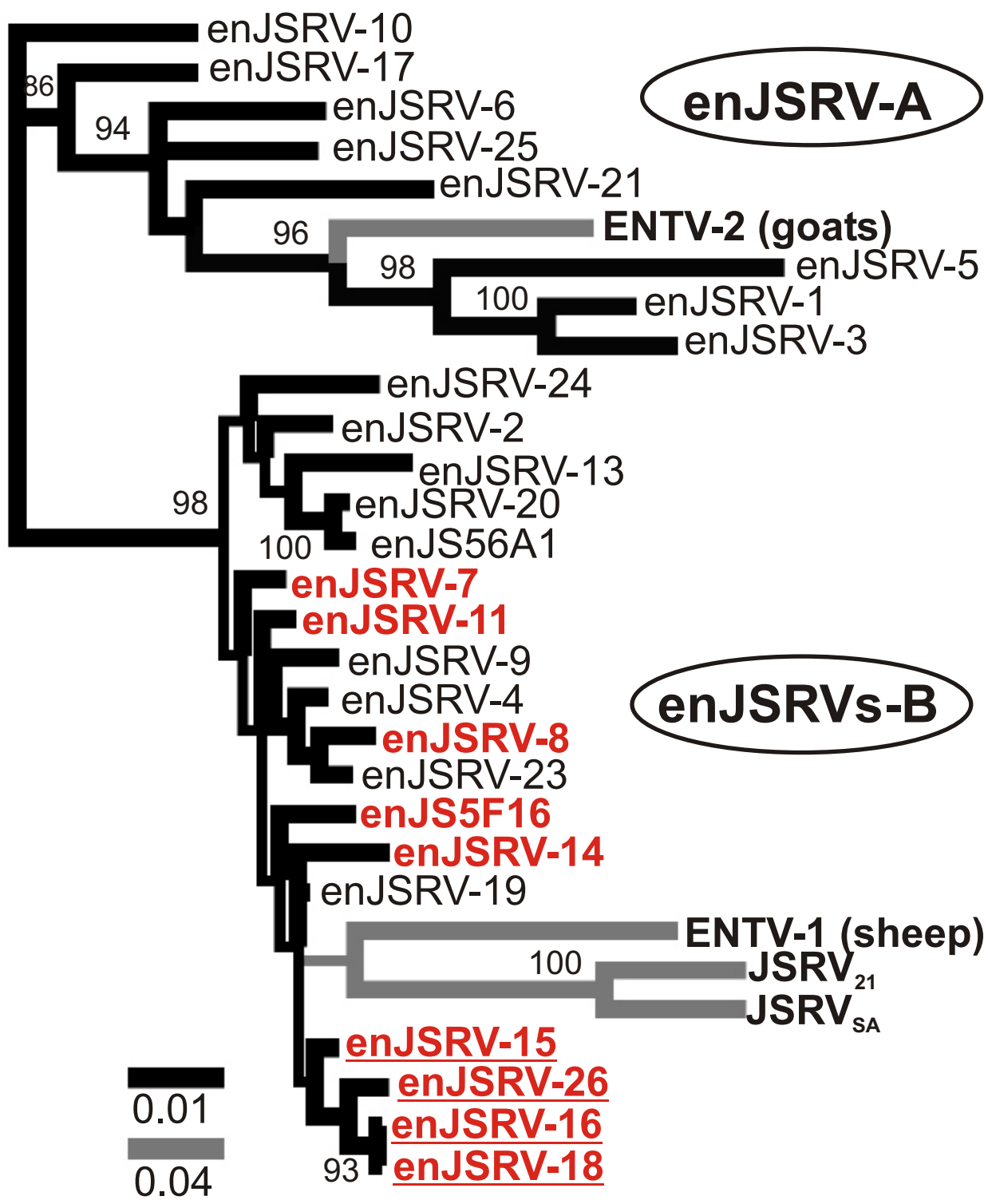

enJSRV-10
enJSRV-17
86
94
enJSRV-6
enJSRV-25
enJSRV-21
96
ENTV-2 (goats)
98
enJSRV-5
100
enJSRV-1
enJSRV-3
enJSRV-A
enJSRV-24
enJSRV-2
enJSRV-13
98
enJSRV-20
100
enJS56A1
enJSRV-7
enJSRV-11
enJSRV-9
enJSRV-4
enJSRVs-B
enJSRV-8
enJSRV-23
enJS5F16
enJSRV-14
enJSRV-19
ENTV-1 (sheep)
100
JSRV21
JSRVSA
enJSRV-15
enJSRV-26
enJSRV-16
93
enJSRV-18
0.01
0.04
