# Supplementary material for: A Paradigm for Virus–Host Coevolution: Sequential Counter-Adaptations between Endogenous and Exogenous Retroviruses
Source: PLoS Pathog. 2007 Nov 9;3(11):e170. doi: 10.1371/journal.ppat.0030170 (PMC2065879; doi:10.1371/journal.ppat.0030170)
Supplement: Supplementary file 2 [file ppat.0030170.sg002.pdf]

A.

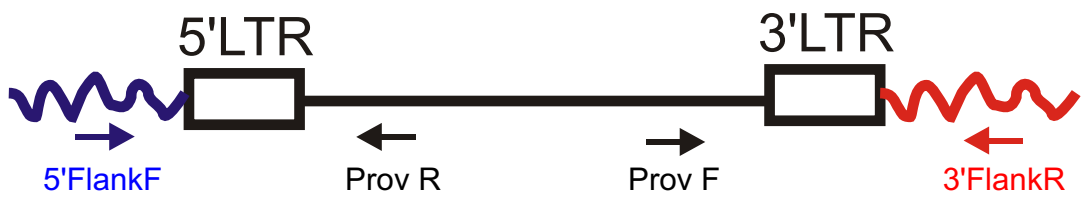

Provirus names	5'FlankF / Prov R	ProvF / 3'FlankR
enJSRV-1	5'-ttgggctgccatggagcgttca / 5'-actgtcgaacagccccgcgttgg	5'-agccttcattcactgtggcga / 5'-agttctggtcagggtgccacctg
enJSRV-2	5'-agcacaaccaaacccagaaccct / 5'-agagtagtggcgaggaaagc	5'-agccttcattcactgtggcga / 5'-gcaggtggaaaagggggcaac
enJSRV-3	5'-acttaattgtgggtcaccсctgg / 5'-ggtgaggaaaactgtcgaacagc	5'-agccttcattcactgtggcga / 5'-actaaggagtccacacgcctaga
enJSRV-4	5'-agcataaccaacgcaatggac / 5'-atcatgggactttcgggctgc	5'-agccttcattcactgtggcga / 5'-ggtggagaaactgtagacttc
enJSRV-5	5'-gtgttgtcatagacacagtgggt / 5'-actgtcgaagtcgagcccca	5'-agccttcattcactgtggcga / 5'-actatacacgtgacatcgcagc
enJSRV-6	5'-ccagttccagaagggaagggag / 5'-agccctacaacctgggttgccca	5'-agccttcattcactgtggcga / 5'-caggggaataactggtgctacct
enJSRV-7	5'-tgtgcacacgtggtgggagtc / 5'-gtagtggcgaggaaaactgtcgagc	5'-agccttcattcactgtggcga / 5'-aggaactccagggtcgtgcca
enJSRV-8	5'-tcagtggatcaatggtctgcga / 5'-gtagtggcgaggaaaactgtcgagc	5'-agccttcattcactgtggcga / 5'-gttatgggatttggggaaaagc
enJSRV-9	5'-agctagcagttttcccagtcacg / 5'-gtagtggcgaggaaaactgtcgagc	5'-agccttcattcactgtggcga / 5'-agactgcatagaacaaaggctgc
enJSRV-10	5'-agcgtggaaatctacccagaagc / 5'-gtagtggcgaggagaactgtcgagc	5'-agccttcattcactgtggcga / 5'-cccattgggatgtggttccgtg
enJSRV-11	5'-tgttgcttcctggcccgcatac / 5'-gtagtggcgaggaaaactgtcgagc	5'-agccttcattcactgtggcga / 5'-gctggttttagaaaaggcagagg
enJSRV-13	5'-gcatttcattcgtaccgctacca / 5'-gtagtggcgaggagaactgtcgagc	5'-agccttcattcactgtggcga / 5'-aggtgtccttacttcaagcctgc
enJSRV-14	5'-catcccacccccagcctctggca / 5'-gtagtggcgaggaaaactgtcgagc	5'-agccttcattcactgtggcga / 5'-ggagttcactcagactcacgtcc
enJSRV-15	5'-cttctagtacagaatatagtggg / 5'-gtagtggcgaggaaaactgtcgagc	5'-agccttcattcactgtggcga / 5'-tcactgttgcttcctgaccagg
enJSRV-16	5'-tgctcagtttcccaggtgcccca / 5'-gtagtggcgaggaaaactgtcgagc	5'-agccttcattcactgtggcga / 5'-ggccagttgacatctgccagga
enJSRV-17	5'-gtagtggcgaggaaaactgtcgaac / 5'-gtagtggcgaggaaaactgtcgaag	5'-agccttcattcactgtggcga / 5'-acttgggttgcttccatgctgt
enJSRV-18	5'-gggaagattcgtttcttaggcgctc / 5'-gtagtggcgaggaaaactgtcgagc	5'-agccttcattcactgtggcga / 5'-caagtgccagagcccagagcca
enJSRV-19	5'-aggctgtatattgtcaccctgc / 5'-gtagtggcgaggagaactgtcgagc	5'-agccttcattcactgtggcga / 5'-tcttccatgaagtgatgggacc
enJSRV-20	5'-agccttcattcactgtggcga / 5'-gtagtggcgaggagaactgtcgagc	5'-agccttcattcactgtggcga / 5'-aggcaagcttgagcatcgtcc
enJSRV-21	5'-tctgtgtttcttggacttgggt / 5'-actgttgaactcgagccccacg	5'-agccttcattcactgtggcga / 5'-ggacaatctcagagacctccagg
enJSRV-22	5'-gcagtctgccaggctcctctg / 5'-gtagtggcgaggaaaactgtcgagc	5'-agccttcattcactgtggcga / 5'-ccatatgtagggattggggggtg
enJSRV-23	5'-gtgatgtgtctcacactgcctgg / 5'-gtagtggcgaggaaaactgtcgagc	5'-agccttcattcactgtggcga / 5'-actgcaaccctgggcaggggcac
enJSRV-24	5'-acaggcaactttggcctggga / 5'-ccccatattagtacagggtctgg	5'-agccttcattcactgtggcaa / 5'-tccaggatgtctggccgtaggtg
enJSRV-25	5'-cctctgtgctaagtgacccacg / 5'-gtagtggcgaggaaaactgtcgaac	5'-agccttcattcactgtggcga / 5'-gtgcaatctataccatgtccca
enJSRV-26	5'-acgcagacaactggccagagtgc / 5'-actgtaccttctctggggaacc	5'-agccttcattcactgtggcga / 5'-ggtggccttacagacgtctcc
enJS56A1	5'-tcatggggtgttcaggagcca / 5'-gtagtggcgaggagaactgtcgagc	5'-agccttcattcactgtggcga / 5'-agcttgagcatcgtccacaca
enJS59A1	5'-gtcagaaaccttacctgcctgag / 5'-gtagtggcgaggaaaactgtcgag	5'-taattgctgtgggcgcggttc / 5'-attcgctagggcgtgctggac

B.

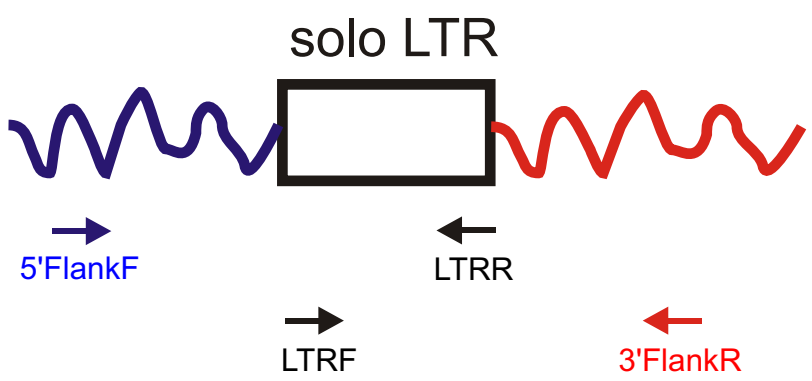

provirus names	5'FlankF / LTRR	LTRF / 3'FlankR
enJSRV-7	5'-tgtgcacacgtggtgggagtc / 5'-gccagcacaagcaagagtcg	5'-gccagcacaagcaagagtcg / 5'-aggaactccagggtcgtgcca
enJSRV-8	5'-tcagtggatcaatggtctgcga / 5'-agagtcacacctgcacagggag	5'-gccagcacaagcaagagtcg / 5'-gttatgggatttggggaaaagc
enJSRV-11	5'-tgttgcttcctggcccgcatac / 5'-gccagcacaagcaagagtcg	5'-gccagcacaagcaagagtcg / 5'-gctggttttagaaaaggcagagg
enJSRV-15	5'-cttctagtacagaatatagtggg / 5'-gccagcacaagcaagagtcg	5'-gccagcacaagcaagagtcg / 5'-tcactgttgcttcctgaccagg
enJSRV-16	5'-tgctcagtttcccaggtgcccca / 5'-gccagcacaagcaagagtcg	5'-gccagcacaagcaagagtcg / 5'-ggccagttgacatctgccagga
enJSRV-18	5'-gggaagattcgtttcttaggcgctc / 5'-gccagcacaagcaagagtcg	5'-gccagcacaagcaagagtcg / 5'-caagtgccagagcccagagcca
enJSRV-22	5'-ggataagctacactataaaaccaaag / 5'-agagtcacacctgcacagggag	5'-gccagcacaagcaagagtcg / 5'-ccatatgtagggattggggggtg
enJSRV-26	5'-acgcagacaactggccagagtgc / 5'-agagtcacacctgcacagggag	5'-gccagcacaagcaagagtcg / 5'-attcgctagggcgtgctggac

C.

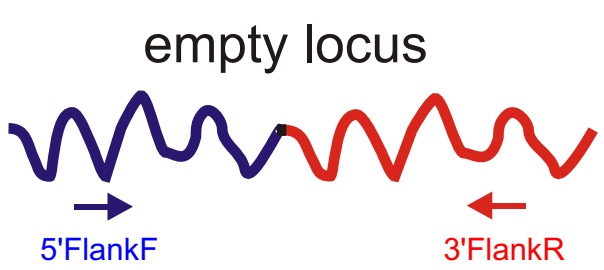

provirus names	5'FlankF / 3'FlankR
enJSRV-7	5'-tgtgcacacgtggtgggagtc / 5'-actcgagaggaagcacgagggtc
enJSRV-8	5'-tcagtggatcaatggtctgcga / 5'-tgggtgagcatgcacacacg
enJSRV-11	5'-tgttgcttcctggcccgcatac / 5'-gctggttttagaaaaggcagagg
enJSRV-15	5'-gtgggaagaaattgcttggtacac / 5'-ccataagggtggtatcgtctgca
enJSRV-16	5'-tgctcagtttcccaggtgcccca / 5'-gtgcaagagctagagctggaagg
enJSRV-18	5'-gggaagattcgtttcttaggcgctc / 5'-atgaaccggactccatggcag
enJSRV-22	5'-ggataagctacactataaaaccaaag / 5'-ccatatgtagggattggggggtg
enJSRV-26	5'-acgcagacaactggccagagtgc / 5'-attcgctagggcgtgctggac
