# Supplementary material for: A Paradigm for Virus–Host Coevolution: Sequential Counter-Adaptations between Endogenous and Exogenous Retroviruses
Source: PLoS Pathog. 2007 Nov 9;3(11):e170. doi: 10.1371/journal.ppat.0030170 (PMC2065879; doi:10.1371/journal.ppat.0030170)
Supplement: Supplementary file 3 [file ppat.0030170.st001.doc]

**Supplemental table 1. Estimated integration time for each enJSRV provirus based on the differences between the 5’ and 3’ LTR.**

| Provirus* | LTR length | nt differences | Estimated Integration (MYA)^§^ |
| --- | --- | --- | --- |
| **enJSRV-26** | 413 | 0 | < 0.45 |
| **enJSRV-15** | 447 | 0 | < 0.45 |
| **enJSRV-16** | 446 | 0 | < 0.45 |
| **enJSRV-18** | 446 | 0 | < 0.45 |
| enJSRV-11 | 447 | 0 | < 0.45 |
| enJSRV-19 | 446 | 0 | < 0.45 |
| enJSRV-8 | 446 | 0 | < 0.45 |
| enJS5F16 | 446 | 0 | < 0.45 |
| enJSRV-20 | 446 | 1 | 0.4 - 0.9 |
| enJSRV-14 | 446 | 1 | 0.4 - 0.9 |
| enJS56A1 | 446 | 2 | 0.9 - 1.9 |
| enJSRV-21 | 446 | 3 | 1.3 - 2.9 |
| enJSRV-6 | 444 | 3 | 1.3 - 2.9 |
| enJS59A1 | 429 | 4 | 1.9 - 4.0 |
| enJSRV-25 | 446 | 4 | 1.8 - 3.9 |
| enJSRV-10 | 444 | 5 | 2.3 - 4.9 |
| enJSRV-2 | 446 | 5 | 2.2 - 4.9 |
| **enJSRV-7** | 446 | 5 | 2.2 - 4.9 |
| enJSRV-1 | 404 | 6 | 3.0 - 6.5 |
| enJSRV-9 | 446 | 7 | 3.1 - 6.8 |
| enJSRV-13 | 446 | 8 | 3.6 - 7.8 |
| enJSRV-5 | 441 | 8 | 3.6 - 7.9 |
| enJSRV-3 | 403 | 8 | 4.0 - 8.6 |
| enJSRV-23^+^ | n.a.^‡^ | n.a. | n.a. |
| enJSRV-17^+^ | n.a. | n.a. | n.a. |
| enJSRV-4^+^ | n.a. | n.a. | n.a. |
| enJSRV-24^+^ | n.a. | n.a. | n.a. |

* Proviruses with an intact genomic organization are shown in bold.

^§^ Millions years ago

^+^These loci lack either an intact LTR or the entire LTR (see Fig. 1).

‡not applicable.
